# A novel dataset of Gupta archer type coins for machine learning-based classification

**DOI:** 10.1016/j.dib.2024.110934

**Published:** 2024-09-17

**Authors:** Ishtiak Al Mamoon, Zakaria Shams Siam, Abdul Akhir Al Galib, Theophil Dango, Kalin Chakma, Pranto Dev, Rubyat Tasnuva Hasan, Muhammad E.H. Chowdhury

**Affiliations:** a Urology and Transplantation Foundation, Bangladesh; b Department of Computer Science, University at Albany, State University of New York, Albany, NY, USA; c Department of Computer Science and Engineering, BRAC University, Dhaka, Bangladesh; d Independent Researcher; e Department of Electrical Engineering, Qatar University, Doha 2713, Qatar

**Keywords:** Numismatics, Gupta archer-type coins, Dataset annotation, Ancient Indian archaeology, Image processing

## Abstract

In the field of numismatics, classifying ancient coins, especially those that have diverse information and cultural heritage is a difficult task. Machine learning algorithms have recently made remarkable advancements in these types of tasks. However, these algorithms largely rely on relevant datasets. This article presents a novel dataset of ancient Gupta archer-type coin images, collected from verified private collections and three popular auction houses with their permission. The images exclusively comprise authentic specimens of ancient Gupta archer-type coins. We aim to establish a reliable resource that adheres to the highest standards of numismatic research. These coins, characterized by their distinctive archer motifs, present a significant challenge in terms of identification due to their scarcity and the intricate nature of their design. To address this, we meticulously curated a dataset by annotating each coin through a combination of visual examination and leveraging insights from numismatic literatures. These coins inherit ancient Indian archaeological insights, and studying these coins could provide insights into ancient Indian archaeology.

Specifications Table

**Table:** 

Subject	Archaeology, history, computer vision, and pattern recognition.
Specific subject area	Numismatic data collection and classification using machine learning, focusing on Gupta archer-type coins.
Types of data	Image, Table
Data format	Raw annotated
Parameters for the data collection	The Gupta archer-type data are .tiff images with a size of 128×256 pixels.
Data collection	The Gupta archer-type coin images are collected from personal collections and three auction houses taking their permission. Collected data are cleaned and processed using vector graphics processing software GNU, Inscape, and PIX. Data analysis is done using a combination of visual examination and leveraging insights from numismatic literatures.
Data source location	All the images are either taken from private collection or from three auction houses that have granted permission to use their coins. The data are validated by Ishtiak Al Mamoon, Life Member (BNCS-341), Bangladesh Numismatic Collectors Society (BNCS).
Data accessibility	Repository name: Dataset-of-Gupta-Archer-Type-Coins-with-Annotations Direct URL to data: https://github.com/Dataset-of-Ancient-Coins/Dataset-of-Gupta-Archer-Type-Coins-with-Annotations

## 1 Value of the Data

• This dataset consists of rare valuable coins of Gupta archer type.
• This dataset can be useful for the study of ancient Indian numismatics and archaeology, especially the archer-type Gupta specimens.
• This dataset can be utilized to develop machine learning models for various purposes such as Gupta archer type coin classification and forgery detection.
• Numismatists can study and explore the provided dataset to find valuable insights.

## 2 Background

The motivation behind compiling this dataset stems from a dual commitment to preserving cultural heritage and advancing numismatic research [1]. Theoretical and methodological considerations were rooted in the fusion of traditional numismatic expertise and contemporary machine-learning techniques [2,3]. The novel dataset was meticulously crafted to serve as a comprehensive resource for the machine learning-based classification [4] modeling of Gupta archer type coins. By anchoring the data generation process in both visual examination and insights [4] drawn from numismatic literatures, we aim to provide a novel, comprehensive dataset for the development and refinement of classification models [5]. This compilation holds significance within the broader context of historical research, contributing to a nuanced understanding of ancient Gupta archer type coins. The datasetʼs theoretical underpinning lies in the integration of domain knowledge with cutting-edge machine-learning methodologies [6]. As a standalone data article, it adds intrinsic value by offering a curated collection of genuine coins, facilitating diverse avenues of exploration for researchers interested in Gupta history, archaeology, and the application of machine learning to numismatics [8]. This dataset serves as a vital reference, aligning with the ethos of preserving and promoting cultural heritage while pushing the boundaries of scholarly inquiry.

## 3 Data Description

The creation of the dataset holds paramount importance for numismatics and archaeology, focusing on the accurate recognition of ancient coin denominations. This dataset, detailed in this article, comprises 507 RGB color images, spanning eighteen (18) distinct classes [7]. All the images are in .tiff format.

The criteria for including images in the dataset are that the coins must belong to the Gupta dynasty, as identified by [1] and numismatic experts. This dataset focuses specifically on archer-type coins from that dynasty, which have distinctive motif features compared to other coin types from the same period. The primary inclusion criterion is the presence of these motifs, such as the king standing with a bow in his left arm and, occasionally, an arrow in his right arm. Additionally, the coins bear Brahmi script inscriptions of the reigning king's name, such as ʻChandraʼ for Chandra Gupta or 'Ku' for Kumara Gupta.

Images were excluded from the dataset if they depicted identified Gupta coins of different types. All non-archer-type Gupta coins were excluded to ensure the dataset only consists of Gupta archer-type coins.

For improving accuracy in machine learning-based model training, the coin images were cleaned, and the background of the images was removed for consistency throughout the images [7]. Table 1 provides a comprehensive overview of the Gupta archer type coin dataset, including the acronyms and the respective image counts for each acronym.

**Table 1:** Description of Gupta archer type coin dataset.

SN	Denomination considered	No. of images in each denomination	Acronyms
1	Budhagupta	5	BG
2	Chandragupta I	25	CG1
3	Chandragupta II	237	CG2
4	Chandragupta III	17	CG3
5	Ghatotkachagupta	2	GKG
6	Jayagupta	7	JG
7	Kumaragupta I	62	KG1
8	Kumaragupta II	7	KG2
9	Kumaragupta III	7	KG3
10	Later Gupta and Hunic King	7	LGHK
11	Narasimhagupta I	19	NG1
12	Narasimhagupta II	5	NG2
13	Parakramaditya	3	P
14	Samacharadeva	4	SD
15	Samudragupta	39	SG
16	Skandagupta	44	SKG
17	Vainyagupta	3	VYG
18	Vishnugupta	14	VG
**Total No of Images**		507	

One of the prime significances of the present study is to facilitate machine learning-based classification tasks using the provided dataset, exploring single and multiple machine learning models along with variations in input image resolution. For instance, optimal image resolution significantly impacts the performance of neural networks for diverse image-processing tasks [6]. Original images, captured at varying resolutions pose challenges for training with deep learning techniques due to large sizes and associated memory constraints [9].

**Fig. 1:**
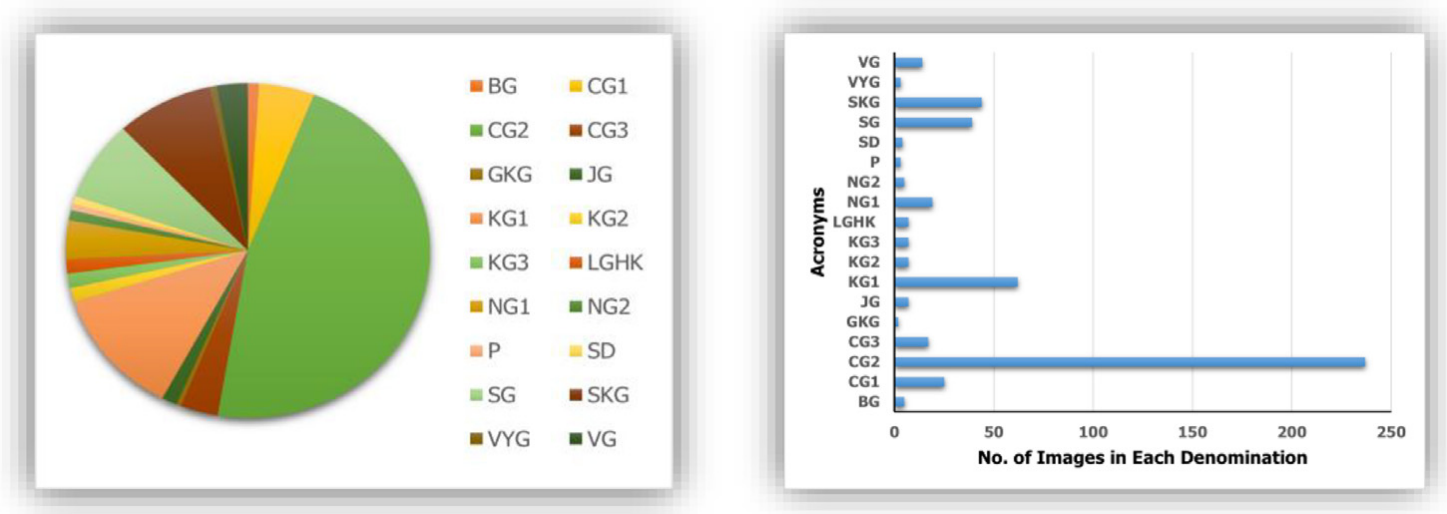
Distribution of each coin denomination and number of images per class in the Gupta archer type coin dataset.

In this dataset, an image consists of both the obverse and reverse of a coin as a rectangular shaped image. To mitigate computational complexity and time constraints, we downscaled the rectangular images to 128 × 256 pixels, stored as .tiff files, which offers a practical compromise between model training time and computational demands. Fig. 1 visually represents the distribution of each denomination and number of images per class in the dataset.

**Fig. 2:**
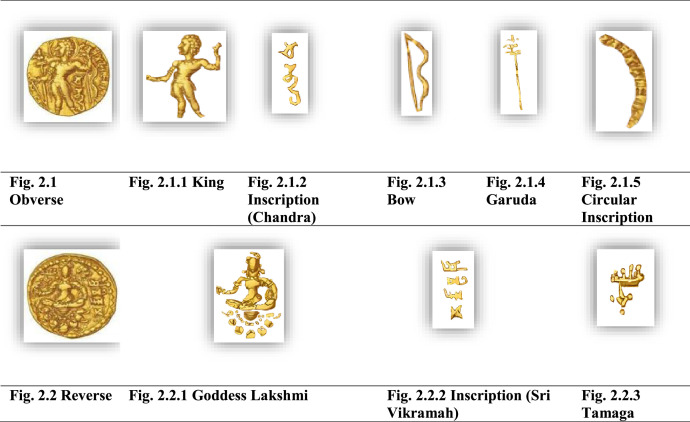
Important features of a Chandragupta III coin.

Fig. 2 shows the important features from both obverse and reverse of a sample Gupta archer type coin image, specifically, a Chandragupta III coin. It is noted that machine learning models should be trained to learn these significant features of a coin to recognize or classify that coin automatically from the input image.

In Gupta archer type coins’ obverse side, the king is generally depicted facing left, standing bare-chested or in Kushan-style clothing along with a bow in his left hand and an arrow in his right hand. ‘Chandra’ is inscribed under left arm (with a circular inscription: 'Deva Sri Maharajadhiraja Sri Chandragupta' and Garuda standard). On the reverse side, Goddess Lakshmi is observed seated on a lotus, with the inscription ʻSri Vikramaʼ.

## 4 Experimental Design, Materials and Methods, Performance Evaluation and Error Analysis

### 4.1 Experimental design

Out of 507 images, 218 images are from our private collection. A total of three popular auction houses, namely Marudhar Arts, Oswal Auction, and Classical Numismatic Gallery, have granted permission to use 186, 52, and 51 images, respectively. As per their directions, we have added ‘© Marudhar Arts’, ‘© Oswal Auction’, and ‘© Classical Numismatic Gallery’ to the filenames of the images collected from Marudhar Arts, Oswal Auction, and Classical Numismatic Gallery, respectively. All the images collected from private collections are captured using ONEPLUS 8T. Camera specification is specified in Table 2. Collected images are separated according to their respective classes and subclasses. Annotation of the coins are done on Google spreadsheet. Finally, an excel file has been saved in the root directory.

**Table 2:** Camera specification.

Device	Description
Camera	ONEPLUS 8T
Type	Smartphone
Megapixel	16MP

The coin acquisition process began in April 2023 and continued until November 2023, primarily during the night. The annotation process commenced in November 2023 and was completed by April 2024. Table 3 provides a detailed description of dataset acquisition (tasks and timeline).

**Table 3:** Data acquisition: Tasks and timeline.

SN	Task	Duration
1	Capture and collection of images	April 2023–November 2023
2	Image cleaning and preprocessing	November 2023–December 2023
3	Removal of background and image resize	January 2024–February 2024
4	Image annotation	January 2024–April 2024

### 4.2 Materials and methods

Original images were captured at varying resolutions, such as, (505 × 277), (365 × 212), (324 × 177), (483 × 278), and (345 × 221) along with different backgrounds. The background adjustment and image resizing were performed using a Python script. The resized image dimension is 128 × 256 pixels (for both obverse and reverse) for all the images. The choice to reduce the photos to dimensions of 128 × 256 pixels was made due to the composition of the raw photographs. These images contained both the front and back sides of each coin in a single rectangular shape. In order to train and test the machine learning models, we divided the photographs into two independent categories: one for the reverse side and one for the obverse side. Each image was thus reduced to a dimension of 128 × 128 pixels. This strategy enables us to retain the distinct characteristics of both aspects of the situation while simultaneously achieving a feasible level of resolution that strikes a compromise between recognizing details and ensuring computing efficiency. The background of all the images was removed and replaced with white color only.

**Fig. 3:**
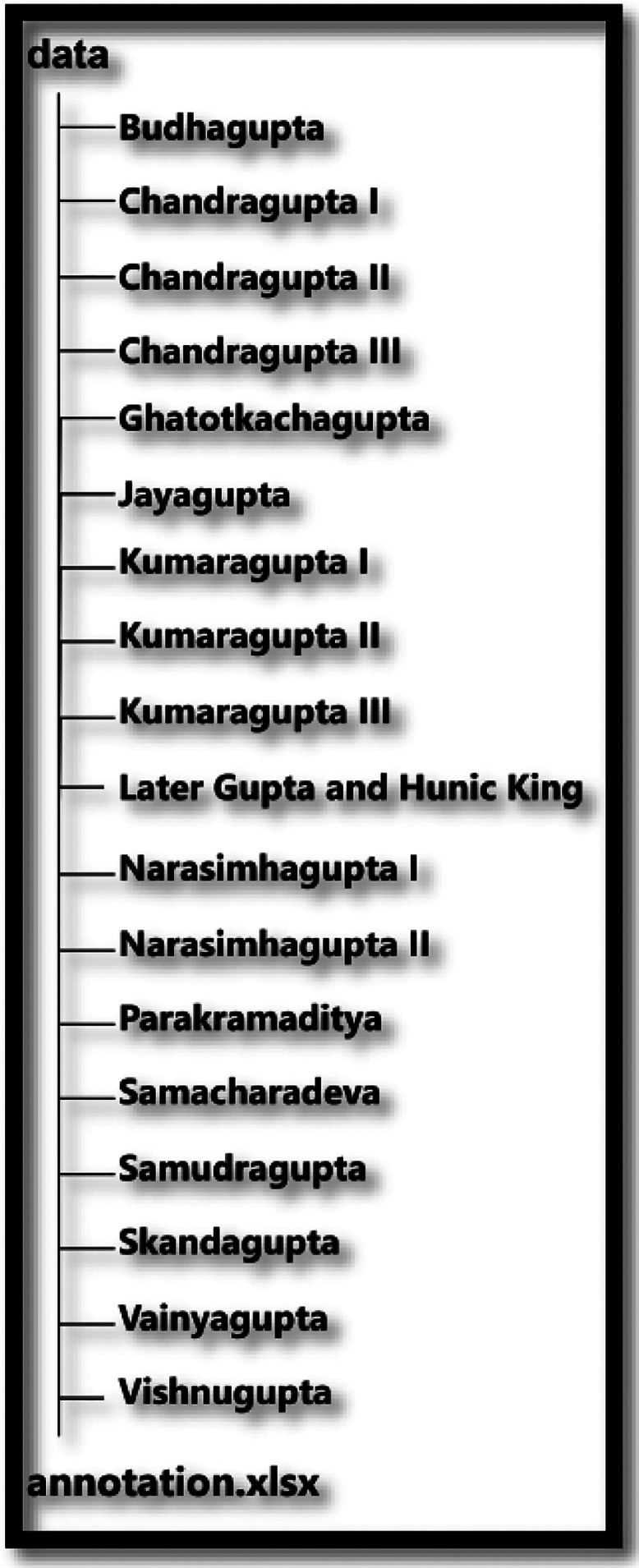
Structure of Gupta archer type coin dataset.

**Fig. 4:**
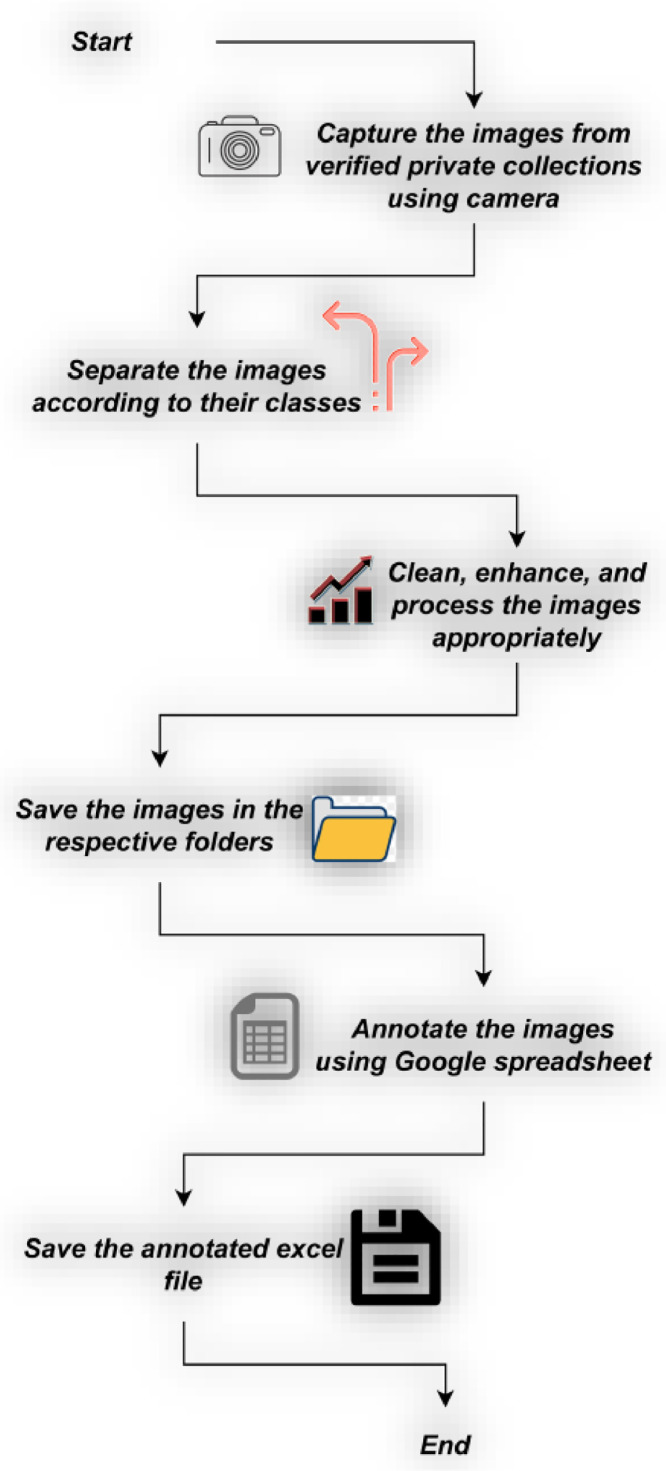
Flowchart of Gupta archer type coin dataset acquisition process from private collection. Other images are collected from three auction houses that have granted permission to use their coins.

Fig. 3 delineates the structure of Gupta archer type coin dataset, whereas Fig. 4 shows the flowchart of the data acquisition process.

We labeled and annotated the image files in the dataset according to [1]. In [1], the author differentiates coins based on the designs of the obverse motif and the weight of the coins. Among these unique designs, there are small variations, referred to as varieties. The collected images from internet sources and personal collections were classified based on [1] and the observations of numismatic experts. Labeling and annotation of the image files were done manually by inserting the annotations into an Excel sheet. Thus, for dataset annotation, we utilized proven archaeological and numismatic sources, and these references offered in-depth knowledge about the historical importance, cultural background, and visual characteristics of every Gupta archer-type coin. By incorporating this academic material, we guarantee that the dataset annotations precisely depict the historical and cultural backgrounds of the coins, thereby upholding a superior level of authenticity and significance in the classification procedure. This approach not only increases the datasetʼs worth for machine learning applications but also guarantees its conformity with the demanding criteria of numismatic research.

### 4.3 Performance evaluation and error analysis

We performed a comprehensive assessment of the machine learning models utilized for classifying Gupta archer-type coins. Our review primarily focused on performance measures including accuracy, precision, recall, and F1-score. The experiments we conducted utilized three distinct deep learning-based ResNet models: ResNet-18, ResNet-50, and ResNet-152. Each model was employed for a binary classification task, specifically to differentiate Gupta archer-type coins from non-Gupta archer-type coins. The ResNet-50 model demonstrated superior performance, with an accuracy of 89.0 %, precision of 88.5 %, recall of 89.2 %, and F1-score of 88.8 %. These results highlight the strength and effectiveness of our approach, as well as the success of our suggested dataset and technique.

In order to guarantee the dependability and applicability of our model, we implemented a thorough validation process. The dataset was divided into three sets: 70 % for training, 20 % for testing, and 10 % for validation. Additionally, we employed 5-fold cross-validation to further assess the models. In order to address the issue of overfitting, a technique called early stopping was employed. This involved stopping the training phase of the model when its performance on the validation set no longer showed any improvement. In addition, data augmentation methods such as random rotations, scaling, and flipping were used to increase the variety of the training data. This helped improve the model's capacity to generalize and identify small differences in coin designs.

Upon doing error analysis, it was found that misclassifications were common in coins that had experienced considerable wear. This was mostly due to the fact that essential distinguishing characteristics, such as the archerʼs bow, were hidden or difficult to discern. Moreover, the use of different minting procedures in different regions resulted in slight variances in design, which presented difficulties for the models. Our study identified three crucial observations: (1) ResNet-50 demonstrated a strong ability to detect well-preserved coins but faced difficulties in identifying worn specimens. This suggests that enhancing data augmentation techniques could enhance the performance of the model. (2) The misclassification issue was partly caused by an imbalance in the distribution of classes, indicating the necessity of either a more balanced dataset or techniques such as oversampling. (3) The significance of having a diverse dataset that encompasses all potential variations within a class was emphasized by the observed regional differences. These observations offer a more profound comprehension of the dataset's usefulness and identify areas that could be enhanced in future research.

We also observe that the effectiveness and generalizability of our coin categorization model are strongly impacted by changes in lighting conditions and angles. Poor lighting can obscure important characteristics such as inscriptions, potentially causing misclassification, while varying angles might distort or conceal elements, making consistent identification difficult. In order to address these problems, our dataset incorporates photographs taken under a wide range of lighting conditions and perspectives, and we utilized data augmentation methods to replicate these changes. This strategy improves the model's capacity to generalize and precisely categorize coins, even when there are variations in lighting and perspective.

## 5 Discussion and Limitations

The dataset we have collected includes all the Gupta archer-type coins that have been published [1]. When considering the suitability of the dataset, we recognize that the quality and diversity of the data are essential for creating strong machine learning models. The dataset we used has 507 photographs distributed over 18 different classes. We took great care in selecting these images to ensure that they cover a wide range of variations and accurately reflect the different classes. Every class depicts unique variations in the design, minting, and wear of the Gupta archer-type coins, offering a wide range of features for the model to acquire knowledge from. Although the total number of photos per class may seem small, the dataset accurately represents the important characteristics and nuanced variations within each class, which are crucial for successful classification. This guarantees that the collection is not only inclusive but also extensive in capturing the intrinsic diversity of these antique coins. Additionally, the dataset was created with the assistance of numismatic specialists, and the photos were obtained from reliable private collections and recognized auction houses, which enhances the credibility and variety of the data. Due to the rarity and historical significance of these coins, we are certain that our dataset, albeit small, is sufficient for training reliable machine learning models capable of reliably categorizing Gupta archer-type coins. Furthermore, we intend to enhance this dataset in future endeavors by incorporating further photos and categories as new specimens become accessible, hence consistently enhancing the resilience of the model.

We utilized authenticated private collections and specifically selected three auction houses—Marudhar Arts, Oswal Auction, and Classical Numismatic Gallery—due to their origin in India and their esteemed reputation for their knowledge in Indian coins. These auction companies primarily specialize in storing Indian coins, in contrast to those that primarily concentrate on foreign currencies, such as Roman coins. By maintaining this focus, we guarantee that our dataset is genuine and directly applicable to the analysis of Gupta archer-type coins.

The dataset, focusing exclusively on Gupta archer type coins, acknowledges inherent challenges posed by the rarity of images. Due to their scarcity, the dataset may not comprehensively represent the entire spectrum of Gupta numismatics. Additionally, the cultural heritage embedded in each coin introduces complexity in classification, as the nuances of artistic variations and historical context may be challenging to be captured fully. While meticulously curated, the datasetʼs scope is confined to Gupta archer type coins, limiting its applicability to a broader numismatic context. Researchers should exercise caution in generalizing findings beyond this specific coin type.

## Ethics Statement

It is noted that all the images are either taken from private collection or from three auction houses mentioned in the manuscript. These auction houses have granted permission to use these coins. It is noted that the data are collected ensuring transparency and adherence to ethical standards.

## Credit Author Statement

**Ishtiak Al Mamoon:** Data curation, Investigation, Supervision, Writing – original draft; **Zakaria Shams Siam:** Supervision, Writing – original draft, Writing – review & editing, Validation; **Abdul Akhir Al Galib:** Methodology, Software, Writing – original draft; **Theophil Dango:** Data curation, Conceptualization, Software, Writing – original draft; **Kalin Chakma:** Data curation, Writing – original draft, Writing – review & editing; **Pranto Dev**: Supervision, Writing – original draft, Writing – review & editing, Validation; **Rubyat Tasnuva Hasan**: Writing – review & editing, Validation; **Muhammad E. H. Chowdhury:** Supervision, Writing – review & editing, Funding acquisition.

## Data Availability

Dataset-of-Gupta-Archer-Type-Coins-with-Annotations (Original data) (GitHub).

## Appendix Supplementary Materials

Image, application 1

Image, application 2

Image, application 3

Image, application 4

Image, application 5
