# Supplementary material for: A novel dataset of Gupta archer type coins for machine learning-based classification
Source: Data Brief. 2024 Sep 17;57:110934. doi: 10.1016/j.dib.2024.110934 (PMC11474177; doi:10.1016/j.dib.2024.110934)
Supplement: Supplementary file 4 [file mmc4.pdf]

Looking for permission and obtaining a copy to publish coin image —non commercial purposes

Dr. Ishtiak Al Mamoon <ishtiakm@pu.edu.bd> Mon, May 13, 2024 at 4:43 PM
To: Marudhar Arts <info@marudhararts.com>
Cc: Zakaria Siam <zakarias@pu.edu.bd>

Attn to :

Maru Rajender
Auctioneer | Editor | Organizer | Director
www.marudhararts.com
Marudhar Arts
85 M. G. Road,
Next to Barton Center,
Bangalore 560 001 (INDIA)

Dear Mr Maru Rajendar,

I hope this message finds you well. I am writing to follow up on the previous permissions granted for the use of images from your collections in our manuscripts on Gupta archer type, Shasanka and samatata data set, which we are preparing to submit to the journal Data in Brief.

We are grateful for your support and the permission you have already granted for the use of these images in our research. However, the journal's requirements for data accessibility and reusability necessitate that we obtain additional permissions. **Specifically, we need to ensure that the images can be freely reused by the readership of Data in Brief.**

Could you kindly extend the permission to include the free reuse of these images by the journal's readership? This would involve allowing the images to be published under terms that permit free access and reuse by others without any restrictions.

We believe that the broader dissemination and potential reuse of these images will greatly benefit the academic community and further highlight the valuable collections held at your institution.

We would greatly appreciate your prompt attention to this matter and your assistance in helping us comply with the publication requirements.

Thank you very much for considering our request. Please let me know if you need any additional information or if there are any issues we can help clarify.

Looking forward to your positive response.
Best Regards,

Ishtiak Al Mamoon, Ph.D., SMIEEE
Associate Professor
Department of Electrical and Computer Engineering
Presidency University, Baridhara Campus
Email: ishtiakm@pu.edu.bd
H/P: +880-171-3229860

[Quoted text hidden]
