# Supplementary material for: A novel dataset of Gupta archer type coins for machine learning-based classification
Source: Data Brief. 2024 Sep 17;57:110934. doi: 10.1016/j.dib.2024.110934 (PMC11474177; doi:10.1016/j.dib.2024.110934)
Supplement: Supplementary file 5 [file mmc5.pdf]

Gmail

Zakaria Shams Siam <zakarias@pu.edu.bd>

Looking for permission and obtaining a copy to publish coin image —non commercial purposes

Marudhar Arts <info@marudhararts.com>
To: "Dr. Ishtiak Al Mamoon" <ishtiakm@pu.edu.bd>
Cc: Zakaria Siam <zakarias@pu.edu.bd>

Wed, May 15, 2024 at 11:23 AM

Dear Sir,

Good morning.

As discussed last time, as long as every image is duly acknowledged whenever it is reused or republished for any purpose with due credits given to each image as - **© Marudhar Arts, India,** you are allowed to use it.

Requesting you to share the Journal which you have mentioned last time.

Thank you

Best Regards
Maru Rajender
Auctioneer | Editor | Organizer | Director

www.marudhararts.com

https://www.facebook.com/MarudharArts/

Marudhar Arts
85 M. G. Road,
Next to Barton Center,
Bangalore 560 001 (INDIA)

+91 - 88670-29800 **(10AM to 6PM IST)**

[Quoted text hidden]
