# Supplementary material for: A novel dataset of Gupta archer type coins for machine learning-based classification
Source: Data Brief. 2024 Sep 17;57:110934. doi: 10.1016/j.dib.2024.110934 (PMC11474177; doi:10.1016/j.dib.2024.110934)
Supplement: Supplementary file 3 [file mmc3.pdf]

Looking for permission and obtaining a copy to publish coin image —non commercial purposes

Marudhar Arts <info@marudhararts.com> Mon, Apr 15, 2024 at 3:39 PM
To: "Dr. Ishtiak Al Mamoon" <ishtiakm@pu.edu.bd>
Cc: Zakaria Siam <zakarias@pu.edu.bd>

Subject: Proper Usage of Images from Marudhar Arts® Auctions in Academic Work

Dear Professor,

We are pleased to grant you and your students permission to utilize images from Marudhar Arts® Auctions for academic purposes. We value the contribution of academic research and are glad to support your endeavors.

However, we kindly request that you and your students adhere to the following guidelines to ensure proper usage and crediting of our images:

1. Please use the correct name of our firm, Marudhar Arts® Auctions, along with the copyright logo and city, as mentioned in our previous correspondence. It is crucial to avoid misspelling our name. It has to be strictly used like this - **© Marudhar Arts, India**

2. While we encourage the use of the data we provide, we expect proper credits to be given for our hard work in every research paper where our images are utilized.

By following these guidelines, you contribute to maintaining the integrity of our brand and acknowledge the effort we put into curating and providing valuable data.

Thank you for your understanding and cooperation.

Best Regards
Maru Rajender
Auctioneer | Editor | Organizer | Director

www.marudhararts.com

https://www.facebook.com/MarudharArts/

Marudhar Arts
85 M. G. Road,
Next to Barton Center,
Bangalore 560 001 (INDIA)

+91 - 88670-29800 **(10AM to 6PM IST)**

[Quoted text hidden]
